# Intensivist performed compression ultrasound (IPCUS) for screening and early detection of deep venous thrombosis in trauma intensive care unit: a prospective study

**DOI:** 10.1007/s00068-025-02920-9

**Published:** 2025-07-04

**Authors:** Ahmad Kloub, Nuri Abdurraheim, Ahmed Ramzee, Naushad Ahmad Khan, Mushreq Alani, Suhail Hakim, Zeenat Bakhsh, Ibrahim Taha, Basil Younis, Gustav Strandvik, Tariq Siddiqui, Yousif Ibrahim, Mohamed Nasr, Sherwan Khoschnau, Khalid Ahmed, Ahad Kanbar, Ashok Parchani, Amor Kilani, Ala’ Suliman, Motasem Awwad, Sana’a Odeh, Lady Faye Estalilla, Ana Giurgea, Abdul Nasar Avayil, Jenalyn Salvador, Jennely Dayandante, Greesha Alias, Sandro Rizoli, Ayman El-Menyar, Hassan Al-Thani

**Affiliations:** 1 Hamad Medical Corporation, Doha, Qatar; 2 Hamad Medical Corporation and Weill Cornell Medicine, Doha, Qatar

**Keywords:** Venous thromboembolism, Chemoprophylaxis, Trauma, Intensivists, Doppler ultrasound

## Abstract

**Introduction:**

Venous thromboembolism (VTE), including deep vein thrombosis (DVT) and pulmonary embolism (PE), is a critical complication in trauma patients, contributing to considerable morbidity and mortality. Multiple factors in trauma pose a challenge to the early initiation of VTE chemoprophylaxis, thereby underscoring the benefit of early detection strategies for DVT. This study evaluated the feasibility of intensivist-performed Compression Ultrasound (IPCUS) for screening of proximal lower limbs for DVT in the trauma intensive care unit (TICU).

**Methods:**

We conducted a prospective study at a level 1 trauma center from November 2021 to May 2023 to assess the utility of IPCUS at the TICU. The study cohort included 800 multi-trauma patients aged 14 years and older admitted to trauma intensive care and step-down units. IPCUS was performed using a three-point compression technique at multiple post-injury time points. Positive or equivocal findings were validated by duplex ultrasonography (DUS). Patients were followed up for one month after discharge.

**Results:**

The cohort was predominantly male (94.2%), with a mean injury severity score of 20. The overall incidence of VTE within the cohort was 3%. VTE chemoprophylaxis was administered to 60% of patients within the first 48 h. IPCUS identified DVT in 10 patients, with a sensitivity of 89% and specificity of 100%. There was no clinical evidence of missed proximal lower limb DVTs. There was a moderate agreement (Cohen’s Kappa score of 0.46) between IPCUS and radiographer-performed DUS.

**Conclusion:**

IPCUS is a feasible and effective screening method for proximal lower limb DVT in trauma patients with high diagnostic accuracy. Enhanced ultrasonography training for intensivists could further improve screening outcomes, reducing the number of equivocal cases. Larger studies are warranted to confirm these findings and establish standardized training protocols for IPCUS in trauma care settings.

**Supplementary Information:**

The online version contains supplementary material available at 10.1007/s00068-025-02920-9.

## Introduction

Venous thromboembolism (VTE), which includes deep vein thrombosis (DVT) and pulmonary embolism (PE), is a significant contributor to morbidity and mortality after major trauma. [1, 2] Patients with DVT may be asymptomatic. PE, on the other hand, is recognized as the third most common cause of death in trauma patients surviving the initial 24 hours (h). [3]

The reported incidence of VTE in trauma patients varies widely, ranging from 1–58%, in patients who do not receive pharmacological thromboprophylaxis. A recent study by Hamada et al. reported a VTE incidence of almost 30% even with adherence to a protocolized thromboprophylaxis regimen. [1–5] Furthermore, in the absence of prophylaxis, the incidence has been reported to be as high as 80%. [6] Autopsy studies revealed asymptomatic DVT in 65% of patients post-trauma. [7, 8]

The occurrence of VTE is associated with extended hospital and intensive care unit (ICU) stays and increased mechanical ventilation days, with a mortality rate reaching 13%. [1] The significant impact on outcomes of this potentially preventable complication has led to the implementation of stringent prophylaxis protocols advocating for earlier initiation of thromboprophylaxis when clinically feasible. However, in the setting of trauma, VTE prophylaxis is a double-edged sword, with the precision required in the timing of initiation and dosing to balance the scales between thrombosis and bleeding. [8] In addition, an important limitation in polytrauma patients is the limited reliability of clinical examination in assessing for lower limb DVT. [9] This has prompted the implementation and evaluation of screening protocols aimed at early identification of DVT prior to its clinical manifestations. [6, 10–12]

Moreover, recent evidence emphasizes the complexity and variability of VTE risk among trauma patients. A comprehensive meta-analysis by Tran et al. identified several prognostic factors—older age, male sex, obesity, higher Injury Severity Score (ISS), pelvic and lower extremity injuries, and delayed prophylaxis, as independent predictors of VTE. Notably, even a 24–48-h delay in initiating prophylaxis was found to double the risk of VTE, highlighting the need for timely intervention and robust surveillance protocols in trauma settings [13].

The gold standard for diagnosing DVT is contrast venography; however, duplex ultrasonography (DUS) is the modality of choice in routine clinical practice. [14, 15] Practically, implementing routine DUS screening for all trauma patients, particularly in high-volume centers, poses logistical challenges and may impose additional financial burdens. [16] The Critical Care Network of the American College of Chest Physicians (ACCP), in collaboration with La Société de Réanimation de Langue Française (SRLF), has defined minimum standards for intensivists to achieve proficiency in critical care ultrasonography [17]. In 2015, guidelines for the performance of bedside ultrasound in critically ill patients, based on current evidence at the time, recommended that “intensivists can reliably perform a focused screening examination by ultrasound to diagnose lower extremity proximal DVT”, rated as Grade 1B evidence [18].

Two primary methods of compression ultrasound for the detection of DVT have been described: the 2-point which assesses the Common Femoral Vein (CFV) and Popliteal Vein (PV), and the 3-point technique, which includes the Femoral Vein (FV), the latter being more time-consuming. A meta-analysis of 17 studies, including 1337 patients, compared both techniques performed by emergency physicians. Pooled sensitivity, specificity, and false negative rate were comparable. Therefore, they recommended that the 2-point technique be performed by trained emergency physicians [14].

The anatomical location of DVT may significantly influence the subsequent risk of PE, with the proximal thromboses (popliteal, femoral, or iliac veins) associated with a higher risk as compared to the distal sites such as the peroneal, posterior, anterior tibial, and muscular veins. [19] As such, most studies focused on scanning proximal veins. Regarding the imaging of the iliac veins by non-radiologists, scanning is challenging and thus is difficult to comment upon. [20] Pooling the current evidence together, it is apparent that screening compression ultrasound may help identify silent DVT and to avoid its possible complications.

This prospective study aimed to assess both the feasibility and diagnostic performance of screening compression ultrasonography for the early detection of lower extremity deep vein thrombosis (DVT) among trauma patients admitted to the ICU. We specifically evaluated the efficacy of a structured three-point Intensivist Performed Compression Ultrasound (IPCUS) protocol in identifying clinically significant DVT.

## Methods

### Study design and setting

This prospective, observational study was performed at Hamad Trauma Center (HTC). HTC is an academic level 1 trauma center treating all moderate to severe traumatic injuries for the entire population of Qatar (approximately 2.7 million), with an average of 1,800 trauma admissions per year.

### Inclusion criteria

All consecutive patients aged 14 years and older who sustained traumatic injuries and were admitted to monitored beds (Intensive Care Unit and step-down units) at HTC from November 2021 to May 2023.

### Exclusion criteria

Pregnant patients, prisoners, patients with active cancer, and patients who were on anticoagulants at the time of admission were excluded from the study.

### Study outcome

The accuracy of bedside intensivist performed venous ultrasound in the detection of proximal lower limb DVT.

### Study hypothesis

IPCUS is a feasible, accurate tool for detecting proximal lower limb DVT.

### Patient selection, data collection, and screening protocol

Demographics and registry data were prospectively collected at the time of admission and throughout the patient’s hospital stay. A team of trauma surgeons and intensivists, including critical care fellows and specialists trained by a senior consultant, performed IPCUS. The team consisted of one senior surgeon, two trauma surgeons, and four trauma critical care fellows. Among them, four members were certified in point of care ultrasound (POCUS, Canada). Critical care fellows and attending physicians were trained in the three-point technique of compression ultrasound as described by Baker et al. [21]

**Fig. 1:**
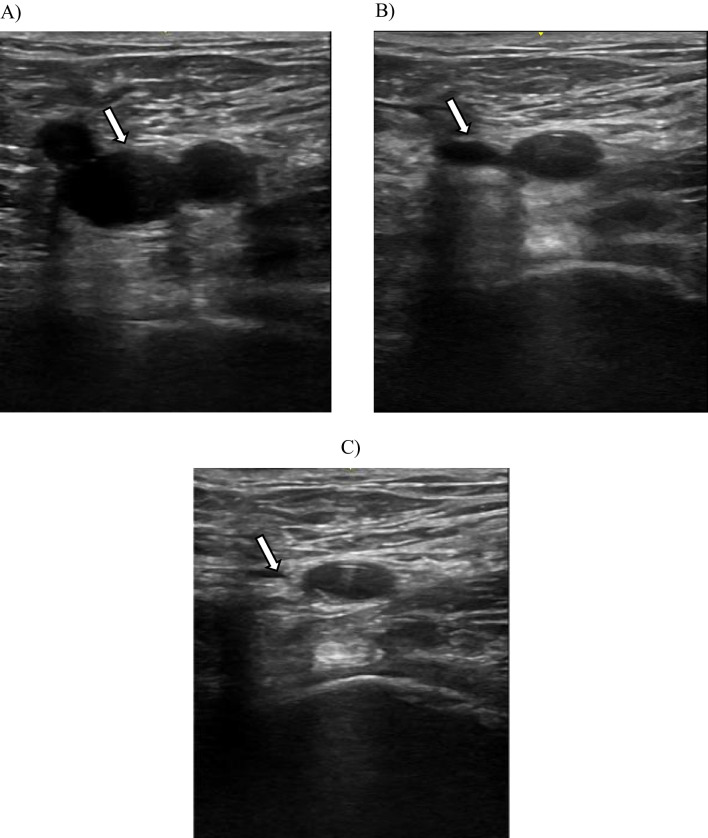
Ultrasound Image by IPCUS. **A**) Normal appearance of left common femoral vein; **B**) Partially compressed left common femoral vein; C) Completely compressed left femoral vein

IPCUS was performed on days 0, 3, 7, 14, 21, and 28 post injury. All IPCUS examinations were conducted using a GE Vscan with Dual Probe machines (GE Healthcare, General Electric Company, USA) equipped with a Linear Probe (3.4–8.0 MHz). The IPCUS technique was performed as follows: after appropriate patient positioning, a transversely oriented ultrasound probe was placed just below the inguinal ligament to visualize the common femoral artery and vein (Fig. 1). The common femoral vein was followed distally, with compression applied at 1-cm intervals until the superficial femoral vein (SFV) was identified. The SFV was then followed by compressing every centimeter sliding down the leg until the SFV dives into the musculature in the mid to distal thigh and is no longer compressible. The popliteal vein was assessed by placing the transducer transversely on the posterior aspect of the knee. It was followed proximally until it was lateral to the artery. Serial compressions every 1 cm were then performed, moving distally until the trifurcation was reached; the study was completed at this point.

Concomitant blood work was taken on days 0, 3, and 7, which included Rotational Thromboelastometry (Rotational thromboelastometry (ROTEM ®, TEM International GmbH, Munich, Germany and TEM Systems, Inc., Durham, NC, USA), D-dimer, Fibrinogen level, Complete Blood Count (CBC), Prothrombin Time (PT), and activated Partial Thromboplastin Time (aPTT).

Screening was discontinued if the patient completed 28 days in the hospital, was discharged, therapeutically anticoagulated, became ambulatory, or deceased.

All screened subjects in the study were placed on appropriate VTE prophylaxis according to the trauma service guidelines. In cases where chemical prophylaxis was contraindicated, mechanical prophylaxis was instituted upon admission in all patients where practically possible.

Any patient found to have a positive or equivocal IPCUS was re-studied by a formal DUS. Similarly, any patient who developed PE had formal imaging to assess for DVT. Briefly, a positive IPCUS was defined as the absolute non-compressibility of any proximal lower limb vein. A negative IPCUS was defined as complete compressibility of all examined proximal lower limb veins. An IPCUS was considered equivocal when the compressibility of one or more veins could not be definitively assessed due to indeterminate or uncertain imaging findings. The patients who were discharged within 28 days were followed up for one-month post-discharge, either in the clinic or by telephone, specifically inquiring about the occurrence of DVT or PE. Outpatient chart reviews were conducted for patients in rehabilitation or long-term care facilities during the first-month post-injury.

In cases where IPCUS was interpreted as negative, patients were continued on standard pharmacologic prophylaxis per institutional protocol. Due to logistical and ethical considerations, formal DUS was not routinely performed for all IPCUS-negative cases. However, a random sample of 50 IPCUS-negative scans was selected for validation with formal DUS to minimize verification bias and assess potential false negatives. These cases were evenly distributed throughout the study period and included patients without clinical signs of DVT.

### Ethical considerations

The study protocol received approval from the Medical Research Center (MRC-01–21–777) at Hamad Medical Corporation (HMC) in Doha, Qatar. Consent was waived for this study since IPCUS, and all blood tests were part of our department's standard care. Nonetheless, patients were provided with a research information sheet detailing the study protocol, the use of information and data collection from the electronic database, and subsequent follow-up procedures.

#### Statistical analysis

Data were expressed as numbers, percentages, and mean ± standard deviation or medians with range whenever appropriate. Demographic, clinical, and laboratory parameters and outcomes were compared among the VTE and non-VTE groups. The chi-square test was performed to analyze differences in categorical variables between groups, and the Fisher exact test was used when observed cell values were less than 5. Continuous variables were compared using Student’s T-test for two groups for parametric data. Mann–Whitney U-test was used for non-parametric data whenever applicable.

Univariate analyses were initially performed to identify variables potentially associated with VTE. Variables with a *p*-value < 0.10 in univariate analysis, along with clinically important variables identified a priori (including Injury Severity Score and presence of vascular injury), were included in a multivariable logistic regression model to determine independent predictors of VTE. Results were expressed as adjusted odds ratios (ORs) with corresponding 95% confidence intervals (CIs).

Agreement between IPCUS and DUS findings was assessed using Cohen's Kappa statistic, which measures interrater agreement beyond chance. Diagnostic accuracy metrics for IPCUS, including sensitivity, specificity, positive predictive value (PPV), and negative predictive value (NPV), were calculated based on the subset of 72 patients who underwent confirmatory duplex ultrasonography (DUS). Equivocal IPCUS results were excluded from the primary diagnostic accuracy calculations. Performance metrics were reported as percentages rounded to the nearest whole number.

Data was expressed as an odds ratio (OR) along with 95% confidence interval (CI). Two-tailed *p* < 0.05 was considered significant. Data was analyzed using the Statistical Package for Social Sciences, software version 21 (SPSS Inc., Chicago, IL).

## Results

**Fig. 2:**
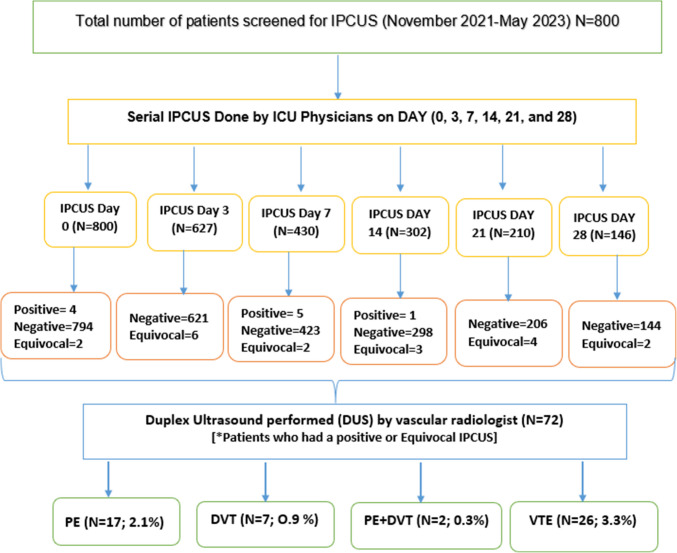
Study flowchart. (PE: Pulmonary Embolism; DVT = deep venous thrombosis; VTE = Venous thromboembolism)

During the study period, 800 patients met the inclusion criteria. The study design is given in Fig. 2. The demographic and clinical characteristics of the cohort are summarized in Table 1. The cohort was predominantly male (94.2%). The most common mechanism of injury was motor vehicle crash (MVC) (45.3%), followed by falls (28.9%). Patients were severely injured, with a mean ISS of 20.

**Table 1:** Demographic and clinical characteristics of the study cohort (*N* = 800)

Variables	Value	Variables	Values
Age	36.8 ± 14.5	**Vascular Injury (*n* = 78; 9.8%)**	
Male	753(94.2%)	Upper Limb	13(16.7%)
Female	46(5.8%)	Lower Limb	31 (39.7%)
BMI (*n* = 786)	26.5 ± 4.9	Thoracic	13(16.7%)
Mechanism of Injury		Neck	13(16.7%)
Motor vehicle crash	362(45.3%)	Abdominopelvic	8 (10.2%)
Pedestrian hit	82(10.3%)	**Ventilatory days (*n* = 345; 43.1%)**	
Fall	231(28.9%)	< 1 days	29 (8.4%)
Fall of Heavy Objects	37 (4.6%)	1 to 5 days	185 (53.6%)
Assault	18 (2.3%)	6–10 days	59(17.1%)
Sports Related	14 (1.8%)	11 to 20 days	47(13.6%)
Others	55 (6.9%)	21 to 30 days	14 (4.1%)
ISS (*n* = 796)	20.1 ± 10.3	> 30 days	11 (3.2%)
Administration of TXA	195 (24.4%)	TICU length of stay (median, range)	3(1–75)
Administration of Fibrinogen	127 (15.9%)	**Discharge Disposition (*N* = 786; 98.3%)**	
Administration of PCC	10 (1.3%)	Home	533 (67.9%)
Severe TBI	356 (44.5%)	Rehabilitation	132 (16.8%)
Chronic heart failure	17 (2.1%)	Long Term Care	31 (3.9%)
Angio-Embolization (*n* = 42; 5.3%)		Transfer to other facilities	44 (5.6%)
Liver	7 (16.6%)	Death	46 (5.8%)
Pelvic	15 (35.7%)		
Splenic	14 (33.3%)		
Others	6(14.4%)		

*BMI* Body Mass Index; *ISS* Injury Severity Score; *PCC* Prothrombin complex concentrate; *TXA* Tranexamic Acid; *TBI* Traumatic Brain Injury; *TICU* Trauma Intensive Care Unit

A significant proportion of patients (44.5%) had severe traumatic brain injury (TBI). Forty-eight (6%) of the cohort had an angioembolization procedure performed mostly for abdominal solid organs or pelvic injuries. Vascular injuries were observed in 78 cases (9.8%), with the lower limb being the most frequently affected region (39.7%). Pharmacological interventions included tranexamic acid (TXA), which was administered to approximately a quarter of the cohort 24.4%). Fibrinogen was administered to 15.9% of patients, while Prothrombin Complex Concentrate (PCC) was used in 1.3%. Ventilatory support was required for 43.1% of patients, with a median duration of 3 days (IQR 1–9). The length of stay in the intensive care unit (ICU) ranged from 1 to 75 days with a median of 3 days, with most patients requiring ventilatory support during their ICU stay. Most patients (67.4%) were discharged home, with 16.8% requiring rehabilitation and 3.9% long-term care. Overall, there were 46 deaths (5.8%).

Table 2 outlines the key laboratory findings and physiological changes observed in trauma patients during hospitalization. Hemoglobin levels showed a marked decline from 13.2 g/dL at baseline to 9.8 g/dL by Day 7. Laboratory tests in the first post-admission week revealed that most patients coagulation parameters, platelet counts, and fibrinogen levels remained within normal limits. Admission metrics revealed mild metabolic acidosis (lactate: 2.4 mmol/L, base excess (BE): −2.5 mmol/L), while 9.3% of patients presented with hypotension. Interventions included transfusion of packed red blood cells (PRBC) (14.5%), fresh frozen plasma (FFP) (5.5%), and platelets (4.3%).

**Table 2:** Laboratory and physiological parameters during hospital stay

Variables	Values
Hemoglobin	
Baseline (*n* = 800)	13.2 ± 4.8
Day 3 (*n* = 631)	10.1 ± 2.1
Day 7 (*n* = 427)	9.8 ± 1.9
Platelets count	
Baseline (799)	245.1 ± 77.2
Day 3 (*n* = 631)	180.5 ± 69.9
Day 7 (*n* = 427)	298.04 ± 111.4
D-Dimer	
Baseline (*n* = 774)	12.9 ± 15.7
Day 3 (*n* = 616)	3.9 ± 7.1
Day 7 (*n* = 418)	5.9 ± 5.2
D-dimer: Fibrinogen Ratio (*n* = 777)	12.73 ± 11.12
International normalized ratio (INR)	
Day 0 (*n* = 800)	1.12 ± 0.15
Day 3 (*n* = 628)	1.11 ± 0.54
Day 7 (*n* = 423)	1.11 ±.0.50
Partial thromboplastin time (PTT)	
Baseline (*n* = 800)	29.08 ± 84.9
Day 3 (*n* = 630)	27.9 ± 6.7
Day 7 (*n* = 425)	27.5 ± 5.5
Fibrinogen Levels at admission (*n* = 783)	2.7 ± 1.7
Lactate at admission (*n* = 772)	2.4 ± 1.9
Base Excess (BE) at admission (*n* = 771)	−2.5 ± 4.5
Fluid Balance within 24 h (*n* = 794)	679.8 ± 1339.7
Systolic Blood Pressure at admission (*n* = 797)	
< 90	74 (9.3%)
≥ 90	723 (90.7%)
Blood transfusion within 24 h	
Packed red blood cells (PRBC)	116(14.5%)
Fresh Frozen Plasma (FFP)	44(5.5%)
Platelets	34(4.3%)
Tranexamic Acid (TXA)	195 (24.5%)
Prothrombin complex concentrates (PCC)	10 (1.3%)
Fibrinogen concentrate	127(15.9%)

In this study, 800 patients underwent serial IPCUS examinations at scheduled time points: baseline (*n* = 800), Day 3 (*n* = 627), Day 7 (*n* = 430), Day 14 (*n* = 302), Day 21 (*n* = 210), and Day 28 (*n* = 146). The number of IPCUS scans decreased over time due to patient discharge, death, or initiation of therapeutic anticoagulation. Across all time points combined, the total number of IPCUS examinations performed was 2515, with a median of 3 IPCUS exams per patient (range 1–6). While IPCUS predominantly showed negative results for thrombotic findings, a small proportion of equivocal or abnormal cases were identified. Follow-up IPCUS evaluations remained consistently negative across time points, indicating a low incidence of significant thrombotic changes. However, the ROTEM assessments revealed a progressive increase in coagulopathy, with abnormalities escalating from 22.1% at baseline to 85.5% by Day 7 (Table 3).

**Table 3:** IPCUS and ROTEM parameters

Variable	Value
Number of IPCUS performed (median; range)	3 (1–6)
Compression Ultrasonography (IPCUS) Baseline (*n* = 800)	
All negative	794(99.3%)
Equivocal	2 (0.3%)
Positive in left Common Femoral vein	1 (0.1%)
Positive in left Femoral vein	2 (0.3%)
Positive in the right Popliteal vein	1 (0.1%)
Compression Ultrasonography (IPCUS) Day 3 (*n* = 627)	
All negative	621 (99.0%)
Equivocal	6(1.0%)
Compression Ultrasonography (IPCUS) Day 7 (*n* = 430)	
All negative	423(98.4%)
Equivocal	2 (0.5%)
Positive in left Common Femoral vein	2 (0.5%)
Positive in the right Common Femoral vein	2 (0.5%)
Positive in the right Femoral vein	1(0.2%)
Compression Ultrasonography (IPCUS) Day 14 (*n* = 302)	
All negative	298 (98.7%)
Equivocal	2(0.7%)
Equivocal left Popliteal vein	1(0.3%)
Positive in the right Common Femoral vein	1(0.3%)
Compression Ultrasonography (IPCUS) Day 21 (*n* = 210)	
All negative	206 (98.1%)
Equivocal	4 (1.9%)
Compression Ultrasonography (IPCUS) Day 28 (*n* = 146)	
All negative	144 (98.6%)
Equivocal	2(1.4%)
Most proximal clotted vein (*n* = 11)	
Left Common Femoral	1(9.1%)
Left Femoral vein	3 (27.3%)
Right Common Femoral	4 (36.4%)
Right Popliteal	1 (9.1%)
Bilateral	1 (9.1%)
Number of IPCUS validated by radiographer	72 (9.0%)

Most patients (59.4%) received chemoprophylaxis within 48 h after admission, while 12.4% received prophylaxis after 72 h. Endotracheal intubation (ETT) within 24 h was most frequent in prehospital settings (20.9%), followed by the trauma room (10.8%) and operating room (6.3%). Central venous access within 24 h predominantly involved jugular (10.4%) and femoral (5.5%) veins. The overall VTE rate was 3.3%, including pulmonary embolism (2.1%) and deep vein thrombosis (0.9%) (Table 4).

**Table 4:** Timing of chemoprophylaxis and procedural characteristics

Variable	Value
Time of initial dose of chemoprophylaxis	*N* (%)
< 24 h	139(17.4%)
24–48 h	336 (42.0%)
48–72 h	172 (21.5%)
> 72 h	99 (12.4%)
Intubation within 24 h	
Operating Room	50 (6.3%)
Prehospital	167(20.9%)
Trauma room	86 (10.8%)
Scene	25 (3.1%)
Trauma intensive care unit	7(0.9%)
Referral to other facility	2 (0.3%)
Surgical Wards	1(0.1%)
Central line within 24 h	
Femoral vein	44 (5.5%)
Juglar vein	83 (10.4%)
Subclavian vein	37(4.6%)
Pulmonary Embolism (PE)	17(2.1%)
Deep Vein Thrombosis (DVT)	7(0.9%)
Both PE and DVT	2(0.3%)
Venous thromboembolism (VTE)	26 (3.3%)

Patients with VTE were compared to patients without VTE in terms of demographic characteristics, clinical parameters, laboratory results, injury profiles, procedural interventions, and outcomes. VTE patients exhibited significantly higher ISS (*p* = 0.001) and greater metabolic derangement upon admission, evidenced by lower base excess levels (*p* = 0.01). Laboratory markers, such as D-dimer, INR, and fibrinogen, did not show statistically significant differences. Procedural interventions were more frequent in the VTE cohort, including central line insertions within 24 h (*p* = 0.04) and a higher prevalence of previous DVT (*p* = 0.04). Although a greater proportion of VTE patients received delayed chemoprophylaxis (after 48 h; 50% vs. 35.9%), this did not reach statistical significance. No significant differences were observed in ICU length of stay or mortality rates (Table 5).

**Table 5:** Comparison between patients with and without VTE

Parameters	No VTE (*n* = 774)	VTE (*n* = 26) (DVT = 7;PE = 17; Both = 2)	OR (95% CI)	*P*-Value
Age	36.9 ± 14.6	35.2 ± 10.4	-	0.53
Gender				
Female	45 (5.8%)	2 (7.7%)	Ref (1)	0.65
Male	729 (94.2%)	24 (92.3%)	0.72 (0.16–3.16)	
BMI	26.4 ± 4.6	29.1 ± 9.1	-	0.16
Baseline blood tests			**-**	
D-Dimer	12.9 ± 11.8	13.9 ± 11.03	-	0.75
Hemoglobin	13.2 ± 4.8	12.5 ± 2.1	-	0.54
Platelets	245 ± 76	242 ± 104	-	0.91
INR	1.10 ± 0.15	1.20 ± 0.17	-	0.10
PTT	29.2 ± 6.6	26.7 ± 7.6	-	0.88
Fibrinogen	2.7 ± 1.7	2.5 ± 0.91	-	0.48
D-dimer: Fibrinogen ratio	11.1 ± 10.1	11.5 ± 9.1	-	0.96
Laboratory parameters at day 3				
D-Dimer	3.9 ± 2.6	3.7 ± 2.0	-	0.91
Hemoglobin	10.1 ± 2.1	9.1 ± 1.6	**-**	0.03
Platelets	181.1 ± 70.1	163.8 ± 62.7	-	0.24
INR	1.1 ± 0.55	1.1 ± 0.10	-	0.86
PTT	27.9 ± 6.8	28.7 ± 5.1	-	0.62
Laboratory parameters day 7				
D-Dimer	5.9 ± 5.2	5.14 ± 3.1	-	0.59
Hemoglobin	9.8 ± 1.9	9.2 ± 1.5	-	0.24
Platelets	299 ± 112	261 ± 66	-	0.23
INR	1.1 ± 0.5	1.2 ± 0.3	-	0.69
PTT	27.5 ± 5.5	26.4 ± 5.2	-	0.45
Lactate at admission	2.5 ± 1.8	3.1 ± 2.7	-	0.09
Base Excess (BE) at admission	−2.5 ± 4.6	−4.6 ± 4.0	**-**	0.01
Fluid Balance within 24 h	511(−4424–10982)	825.5 (−802–7414)	-	0.15
TXA	189(24.5%)	6 (23.1%)	0.92 (0.36–2.34)	0.91
PCC	10 (1.3%)	0 (0.0%)	-	1.000
Fibrinogen therapy	123(15.9%)	4(15.4%)	0.96(0.32–2.83)	1.000
Injury Severity Score (ISS)	19.8 ± 10.2	27.4 ± 10.7	**-**	0.001
ISS Category				
ISS < 16	295(38.3%)	4(15.4%)	Ref (1)	0.01
ISS ≥ 16	475(61.7%)	22(84.6%)	3.41(1.16–10.0)	
SBP at admission				
< 90	68 (8.8%)	5 (19.2%)	Ref (1)	0.08
≥ 90	705 (91.9%)	21(80.8%)	0.40(0.14–1.10)	
Previous DVT	**3 (0.4%)**	1 (3.8%)	10.24(1.02–101.9)	0.01
History of CHF	17 (2.2%)	0 (0.0%)	-	0.69
Severe TBI	344 (44.5%)	12 (46.2%)	1.06(0.482.34)	1.00
Central line within 24 h	154(19.9%)	10(38.5%)	2.51(1.12–5.65)	0.04
Spinal cord Injury	167 (21.6%)	5 (19.2%)	0.86(0.32–2.32)	0.49
Unstable Spinal Fracture	75 (9.7%)	3 (11.5%)	1.21(0.36–4.13)	0.73
Pelvic fracture	150(19.4%)	7 (26.9%)	1.53(0.63–3.70)	0.32
Long bone fracture	165(21.3%)	10 (38.5%)	2.30(1.02–5.17)	0.05
Vascular Injury	73(9.4%)	5(19.2%)	2.28(0.82–6.23)	0.09
LOS in TICU	3(1–75)	5(1–30)	-	0.13
VTE prophylaxis interruptions	2(1–33)	1 (1–7)	-	0.62
Blood transfusion within 24 h				
PRBC	111(14.3%)	5(19.2%)	1.42(0.52–3.85)	0.67
FFP	44(5.7%)	0(0.0%)	-	0.39
Platelet	33(4.3%)	1(3.8%)	0.89(0.11–6.83)	1.000
Prehospital time	94.2 ± 79.4	88.1 ± 66.3	-	0.69
Time of initial dose of chemoprophylaxis				
Early (Within 48 h)	463(64.1%)	12 (50.0%)	Ref (1)	0.19
Late (After 48 h)	259 (35.9%)	12(50.0%)	1.78(0.79–4.03)	
Ventilatory days (VentD)				
< 1 day	27 (4.5%)	2 (8.7%)	Ref (1)	0.49
1 to 5 days	180(30.1%)	5(21.7%)	0.37(0.06–2.03)	
6 to 10 days	57(9.5%)	2 (8.7%)	0.47(0.06–3.54)	
11 to 20 days	43(7.2%)	4 (17.4%)	1.26(0.21–7.3)	
21 to 30 days	14 (2.3%)	0 (0.0%)	-	
> 30 days	11(1.8%)	0(0%)	-	
VentD median&IQR	3(1–9)	5(1–18)	-	0.57
Mortality	45 (5.8%)	1(3.8%)	0.64 (0.08–4.89)	0.67

Abbreviations: *BMI* Body mass index; *INR* International normalized ratio; *PTT* Partial thromboplastin time; *TXA* Tranexamic acid; *PCC* Prothrombin complex concentrates; *DVT* Deep Vein Thrombosis; *CHF* Chronic Heart Failure; *TBI* Traumatic Brain Injury; *LOS* Length of Stay; *PRBC* Packed red blood cells; *FFP* Fresh Frozen Plasma

There were 26 VTEs during hospitalization, resulting in an incidence of 3%. This included 7 DVT, 17 PE, and 2 patients who developed both DVT and PE. The right common femoral vein was identified as the most common site for DVT. 64.1% of patients received the first dose of VTE chemoprophylaxis within 48 h of admission. Notably, early central line insertion, within 24 h, was associated with a higher occurrence of VTE (odds ratio (OR) = 2.51; *p* = 0.02). The femoral site specifically showed an elevated VTE risk (OR 3.33, *p* = 0.02). Timing of initiation of chemoprophylaxis also impacted VTE likelihood; late administration had a higher, albeit not statistically significant, risk than early chemoprophylaxis (OR 1.78, *p* = 0.15). Among vascular injury locations, thoracic injuries were significantly associated with VTE (OR 5.78, *p* = 0.01), and lower limb injuries showed a borderline association (OR 3.47, *p* = 0.04). (Table 6).

**Table 6:** Univariate analysis for the risk prediction of VTE based on central line insertion, time of chemoprophylaxis, and vascular injury

Variables	No VTE (*n* = 774)	VTE (*n* = 26) (DVT = 7; PE = 17; Both = 2)	OR (95% CI)	*P*-value
Central Line first 24 h	154(19.9%)	10(38.5%)	2.51 (1.12–5.65)	0.02
Femoral	40 (5.2%)	4 (15.4%)	3.33(1.09–10.14)	0.02
Juglar	79(10.2%)	4(15.4%)	1.60(0.54–4.76)	0.39
Subclavian	34(4.4%)	2(7.7%)	1.81(0.41–7.99)	0.42
Chemoprophylaxis				
Early	463(64.1%)	12 (50.0%)	1 (Reference)	0.15
Late	259(35.9%)	12(50.0%)	1.78 (0.79–4.04)	
Vascular Injury				
Lower Limb	28(3.6%)	3 (11.5%)	3.47(0.98–12.26)	0.04
Thoracic	11(1.4%)	2(7.7%)	5.78(1.32–30.24)	0.01

Abbreviations: *VTE* Venous thromboembolism; *DVT* Deep vein thrombosis; *PE* Pulmonary Embolism; *OD* Odds Ratio; *CI* Confidence Interval

### Multivariate analysis

Revealed that thoracic vascular injury is associated with significantly increased odds of developing VTE (OR = 10.84; p = 0.006). A higher ISS also significantly correlated with an increased risk of VTE (OR = 1.033; p = 0.02). Other variables assessed in the analysis, including age, gender, baseline hemoglobin levels, base excess, central line placement within 24 h, early chemoprophylaxis, lower limb vascular injury, and long bone fractures, did not demonstrate a significant association with VTE risk (Table 7).

**Table 7:** Multivariate analysis for the predictors of VTE (*N* = 719)

Variables	Odds ratio (OR)	95% Confidence Interval (CI)		*P* value
		Lower	Upper	
Age	0.99	0.966	1.030	0.87
Gender (Male)	1.25	0.214	7.316	0.80
Hemoglobin levels (Baseline)	0.90	0.725	1.124	0.36
Base Excess (Baseline)	0.96	0.906	1.031	0.29
Central line within 24 h (Femoral)	2.23	.548	9.029	0.26
Early chemoprophylaxis	1.43	.579	3.513	0.44
Vascular Injury (Thoracic)	10.84	1.965	59.85	0.006
Vascular Injury (Lower Limb)	0.37	0.083	1.685	0.20
Injury Severity Score (ISS)	1.03	1.006	1.086	0.02
Long bone fracture	0.46	0.172	1.232	0.12
Previous Deep vein thrombosis	0.05	0.006	0.695	0.02

Each IPCUS examination conducted at the scheduled intervals (Days 0, 3, 7, 14, 21, and 28) was considered an independent diagnostic attempt to detect DVT. While 800 patients were enrolled, each patient underwent serial IPCUS scans based on their clinical course and ICU length of stay. Therefore, the total number of IPCUS examinations performed exceeded the number of enrolled patients. The median number of IPCUS scans per patient was 3 (interquartile range [IQR]: 2–5). Not all patients completed scans at six-time points due to early discharge, mortality, or transitions to therapeutic anticoagulation. A total of 10 patients had positive IPCUS: four cases on day 0, five on day 7, and one on day 14. All positive IPCUS exams were validated by a vascular radiographer, along with 14 equivocal IPCUS and 50 negative studies (Table 8). Out of the 50 negative IPCUS, only one was found to be positive in the posterior tibial vein, and one positive DUS in an equivocal IPCUS.

**Table 8:** Validation of IPCUS findings by vascular radiologist (DUS) for the diagnosis of Deep Vein Thrombosis (*N = 72)

IPCUS by ICU physicians	DUS validated by vascular radiographer		Cohen’s Kappa
	Positive (*n* = 10)	Negative (*n* = 62)	
Positive (*n* = 8)	8 (80%)	0 (0%)	0.46**
Negative (*n* = 50)	1 (10%)	49 (80.3%)	
Equivocal (*n* = 14)	1 (10%)	13 (21.3%)	

^*^Out of the 72 cases reviewed and validated by vascular radiologists, there was a moderate level of agreement between ICU physicians and vascular radiologists for the diagnosis of DVT; ** *p* value < 0.001; DUS: Duplex Ultrasound. Kappa values were interpreted as follows: < 0.20 = slight agreement; 0.21–0.40 = fair agreement; 0.41–0.60 = moderate agreement; 0.61–0.80 = substantial agreement; and > 0.80 = almost perfect agreement

The review of 72 cases by vascular radiologists demonstrated a significant level of agreement between IPCUS done by ICU physicians and DUS done by vascular radiologists with a Cohen’s Kappa score of 0.46, indicative of moderate agreement. Overall, after excluding equivocal cases, sensitivity and specificity were noted to be 89% and 100% respectively with a positive predictive value of 100% (Table 9).

**Table 9:** Diagnostic performance of IPCUS (*n* = 58)

Statistic	Value	95% CI
Sensitivity	88.8%	51.7% to 99.7%
Specificity	100.0%	92.7% to 100.0%
Positive Predictive Value	100.0%	63.0% to 100.0%
Negative Predictive Value	98.0%	88.5% to 99.6%
Accuracy	98.2%	90.7% to 99.9%

^*^The analysis accounted only for positive (*N* = 8) and negative cases (*N* = 50), excluding equivocal cases from the study. Sensitivity, specificity, positive and negative predictive value, and accuracy are expressed as percentages

### Follow-up

Almost 80% (*n* = 632) of the patients were followed up (in-person or by telephone) for one month after discharge, among whom two were diagnosed with pulmonary embolism (PE) without DVT, while 122 (15%) of the patients lost follow-up.

## Discussion

In identifying proximal lower limb DVT, this study showed significant concordance between IPCUS performed by trauma intensivists and DUS performed by vascular radiologists. The only missed thrombus was in the posterior tibial vein, which is technically below the knee, yet we chose to include this case because the patient had a concomitant PE. Excluding the equivocal cases, our results showed a sensitivity of nearly 89% and a specificity of 100%. These findings were consistent with results from other studies that support the use of routine screening for IPCUS. The current investigation, to our knowledge, is the first prospective observational study of DVT screening by trauma ICU physicians with no or limited previous experience in DVT ultrasound in the Middle East region.

Most current studies and recommendations do not involve trauma cohorts [17, 18]; however, a preliminary study of intensivist-performed DVT ultrasound screening in trauma ICU patients (APSIT) investigated this specific group prospectively. They included 117 trauma patients who underwent routine IPCUS and duplex ultrasonography (DUS) within 96 h of admission and twice weekly until DVT diagnosis, ICU discharge, or death. They detected 16 patients with DVT. The population was like our study, with the majority being males with traumatic brain injuries and severe ISS (27). Femoral central lines were identified as a major risk factor for the development of DVT. There were 16 cases of proximal lower limb DVTs with an agreement of 96.7% with vascular radiologists performing DUS [6]. These findings, along with ours, corroborate the findings of a multicenter prospective study by Kory et al., which included 128 patients from medical, surgical, and cardiothoracic ICUs. IPCUS was performed on patients who were suspected of having DVT, PE, or both. They reported a sensitivity of 86% and a specificity of 96% with a diagnostic accuracy of 95% [22]. Similarly, Caronia and colleagues described their findings of residents performing IPCUS on 75 patients in medical ICUs. They employed the two-point technique. The results indicated a sensitivity for common femoral and popliteal DVT of 86% and a specificity of 97% [23].

In our analysis, equivocal exams were treated as indeterminate and were excluded from sensitivity/specificity calculations. We acknowledge that this approach could bias diagnostic accuracy estimates upward by effectively analyzing a subset of easier-to-interpret cases and may inflate the sensitivity and specificity. Nevertheless, all equivocal IPCUS results in our study were followed up with confirmatory DUS for patient safety, and one of these equivocal cases was found to have a DVT on DUS. If we were to count that equivocal case as a false-negative IPCUS result (i.e., treating indeterminate as a missed DVT), the calculated sensitivity of IPCUS would decrease from ~ 89% to ~ 83%.

A study by Galien et al. conducted during the COVID-19 pandemic involving ICU residents with minimal to no experience in DVT ultrasonography showed encouraging results. Out of 56 patients admitted to the ICU with COVID-19 infection who were screened for lower limb DVT, 4 tested positive, with three confirmed on formal ultrasound. Importantly, none of the 52 negatively screened patients were detected to have DVT on follow-up [24].

In this study, the incidence of VTE was well below the described range (5–63%) [25–27]. This could be explained by the stringent departmental practice of early initiation of prophylaxis, with more than 60% of patients receiving the first dose within 48 h and 75% within 72 h. Those who received chemoprophylaxis late were more likely to develop VTE (OR 1.9, 0.77–5.15). This is an inherent problem in trauma patients, as early prophylaxis is not always feasible. Silent or partial DVT may have been missed and could have contributed to our low incidence. Additionally, there were no mortalities related to DVT or PE. The risk factors associated with an increased incidence of VTE included higher ISS and thoracic vascular injuries.

Our findings are aligned with recent literature emphasizing patient-specific and modifiable factors contributing to VTE risk in trauma cohorts. A meta-analysis by Tran et al. supports our observation that a higher ISS and delayed chemoprophylaxis are critical risk factors [13]. Furthermore, a systematic review by Bassa et al. underscored global variability in VTE incidence in trauma patients, reporting DVT rates ranging from 0.59% to 57.6% and PE rates between 0.35% and 24%. Surgery, pelvic trauma, and delayed prophylaxis were consistently associated with increased DVT risk [28]. From a diagnostic standpoint, Zaki et al. conducted a meta-analysis on the utility of point-of-care ultrasound (POCUS) for DVT detection. They reported excellent diagnostic performance, with pooled sensitivities of 92.3% and 89.2% and specificities of 96.9% and 92.7% for 2-point and 3-point techniques, respectively [16]. These findings further support our study's rationale for adopting IPCUS as a front-line screening tool, particularly in resource-constrained or high-volume trauma ICU settings, where rapid, bedside decision-making is critical.

Similarly, a meta-analysis by Ratnasekera et al. evaluating active implementation strategies (e.g., clinical alerts, education, and audit-feedback mechanisms) to improve VTE prophylaxis in trauma patients; revealed that while these strategies significantly increased adherence to prophylaxis protocols (OR = 2.94), they did not consistently translate to reduced VTE rates—suggesting a need for multifaceted interventions that go beyond compliance to also address timely risk stratification, education, and detection tools [29].

### Study strength and weakness

One of the strengths of our study was the repeated use of IPCUS as a surveillance tool across multiple time points. Each IPCUS examination was considered a new diagnostic opportunity to identify DVT, allowing for longitudinal thrombotic risk assessment during the ICU stay. All patients underwent screening three times in the first week of admission and weekly thereafter until discharge, death, or completion of 28 days with concomitant laboratory investigations. Although the study enrolled 800 patients, the actual number of IPCUS exams performed (*n* = 2515) was substantially higher due to the serial nature of the screening protocol. This design reflects real-world implementation and demonstrates that IPCUS can be feasibly integrated into trauma ICU workflows for routine bedside DVT screening. However, due to variability in patient length of stay and outcomes, not all patients completed the set of 06 IPCUS studies, which limited complete longitudinal data capture. Moreover, a trained vascular radiologist evaluated any equivocal tests by formal duplex ultrasonography. Close follow-up was carried out till one month post-discharge to assess for missed events.

Obtaining routine DUS for screening as prescribed in our protocol was not logistically feasible. Therefore, DUS was only done for cases with positive and equivocal IPCUS, patients who developed PE, and patients who had clinical suspicion based on physical examination. Consequently, despite negative IPCUS and the absence of clinical signs, subclinical clots may have been overlooked. Another limitation of our study concerns the verification process used to assess IPCUS accuracy. While all IPCUS-positive and equivocal cases underwent confirmatory DUS to ensure diagnostic precision, only a randomly selected subset of IPCUS-negative cases selected based on clinical suspicion (*n* = 50) was validated by DUS. IPCUS-negative cases without confirmatory DUS were assumed to be true negatives. As such, the reported sensitivity and specificity values are derived from this verified subset (*n* = 72) and may not reflect the full cohort. Although this approach aligns with real-world diagnostic workflows and follows best practices for internal validation within resource-limited settings, it introduces the possibility of verification bias, as not all IPCUS-negative results were confirmed. Nonetheless, no patients with negative IPCUS developed clinically apparent proximal DVT during hospitalization or follow-up, lending reassurance to the validity of our screening strategy.

Moreover, IPCUS was performed by a team of trauma surgeons and intensivists, including fellows, specialists, and consultants, most of whom had received informal training in compression ultrasonography. However, there were no clinically significant missed DVT events during hospitalization or the one-month post-discharge follow-up period. Of the 50 negative IPCUS reviewed by a vascular radiologist, only one came out positive, but that too was in the posterior tibial vein, which was beyond the scope of the IPCUS examination. Nonetheless, formalized training of the performer may be beneficial in further optimizing diagnostic accuracy.

## Conclusion

Our findings support the use of intensivist-performed compression ultrasonography for the screening of high-risk patients for DVT. Importantly, no major/clinically significant DVTs were missed. Short-term training in DVT ultrasonography can help trauma surgeons to accurately screen patients. However, as ultrasonography becomes more accessible and portable, increased training and experience may help decrease rates of equivocal studies. The integration of IPCUS into trauma ICU workflows offers a feasible, accurate, and resource-efficient approach to enhance DVT screening. When complemented with risk-based prophylaxis strategies and implementation support, IPCUS could contribute meaningfully to improving VTE prevention efforts, especially in settings where radiology access may be delayed or limited. Largerscale studies will be required to validate this modality further and create standards for training intensive care physicians in this relatively simple but effective screening tool.

## Supplementary Information

Below is the link to the electronic supplementary material.

Supplementary file1 (DOCX 20 KB)

## Data Availability

All data, figures, and tables are presented in the manuscript. It will be available at reasonable request and after approval of the medical research center of Hamad Medical Corporation after signing a data-sharing agreement form.
