# Supplementary material for: Intensivist performed compression ultrasound (IPCUS) for screening and early detection of deep venous thrombosis in trauma intensive care unit: a prospective study
Source: Eur J Trauma Emerg Surg. 2025 Jul 4;51(1):245. doi: 10.1007/s00068-025-02920-9 (PMC12227448; doi:10.1007/s00068-025-02920-9)
Supplement: Supplementary file 1 — Supplementary file1 (DOCX 20 KB) [file 68_2025_2920_MOESM1_ESM.docx]

| **Suppl Table 1: Demographic and clinical characteristics of the study cohort (N=800)** | | | |
| --- | --- | --- | --- |
| **Variables** | **Value** | **Variables** | **Values** |
| Age | 36.8±14.5 | **Vascular Injury (n=78 ; 9.8% )** | |
| Male | 753(94.2%) | Upper Limb | 13(16.7%) |
| Female | 46(5.8 %) | Lower Limb | 31 (39.7%) |
| BMI (n=786) | 26.5±4.9 | Thoracic | 13(16.7%) |
| **Mechanism of Injury** |  | Neck | 13(16.7%) |
| Motor vehicle crash | 362(45.3 %) | Abdominopelvic | 8 (10.2%) |
| Pedestrian hit | 82(10.3 %) | **Ventilatory days (n=345; 43.1%)** |  |
| Fall | 231(28.9 %) | <1 days | 29 (8.4%) |
| Fall of Heavy Objects | 37 (4.6 %) | 1 to 5 days | 185 (53.6%) |
| Assault | 18 (2.3%) | 6-10 days | 59(17.1%) |
| Sports Related | 14 (1.8 %) | 11 to 20 days | 47(13.6%) |
| Others | 55 (6.9%) | 21 to 30 days | 14 (4.1%) |
| **ISS (n=796)** | 20.1±10.3 | >30 days | 11 (3.2%) |
| **Administration of TXA** | 195 (24.4%) | TICU length of stay (median, range) | 3(1-75) |
| **Administration of Fibrinogen** | 127 (15.9%) | **Discharge Disposition (N=786; 98.3%)** |  |
| **Administration of PCC** | 10 (1.3%) | Home | 533 (67.9%) |
| **Severe TBI** | 356 (44.5%) | Rehabilitation | 132 (16.8 %) |
| **Chronic heart failure (CHF)** | 17 (2.1 %) | Long Term Care | 31 (3.9%) |
| **Angio-Embolization (n=42; 5.3%)** |  | Transfer to other facilities | 44 (5.6 %) |
| Liver | 7 (16.6%) | Death | 46 (5.8%) |
| Pelvic | 15 (35.7%) |  |  |
| Splenic | 14 (33.3%) |  |  |
| Other | 6(14.4%) |  |  |
| BMI: Body Mass Index; ISS: Injury Severity Score; PCC: Prothrombin complex concentrate; TXA: Tranexamic Acid; DVT: Deep Vein Thrombosis; TBI: Traumatic Brain Injury; TICU: Trauma Intensive Care Unit. | | | |

| **Suppl Table 2: Laboratory and physiological parameters data during hospital stay** | |
| --- | --- |
| **Variables** | **Values** |
| **Hemoglobin** | |
| Baseline (n=800) | 13.2±4.8 |
| Day 3 (n=631) | 10.1±2.1 |
| Day 7 (n=427) | 9.8±1.9 |
| **Platelets counts** |  |
| Baseline (799) | 245.1±77.2 |
| Day 3 (n=631) | 180.5±69.9 |
| Day 7 (n=427) | 298.04±111.4 |
| **D-Dimer** | |
| Baseline (n=774) | 12.9±15.7 |
| Day 3 (n=616) | 3.9±7.1 |
| Day 7 (n=418) | 5.9±5.2 |
| D-dimer: Fibrinogen Ratio (n=777) | 12.73±11.12 |
| **International normalized ratio (INR)** | |
| Day 0 (n=800) | 1.12±0.15 |
| Day 3 (n=628) | 1.11±0.54 |
| Day 7 (n=423) | 1.11±.0.50 |
| Partial thromboplastin time **(PTT)** |  |
| Baseline (n=800) | 29.08±84.9 |
| Day 3 (n=630) | 27.9±6.7 |
| Day 7 (n=425) | 27.5±5.5 |
| **Fibrinogen Levels at admission (n=783)** | 2.7±1.7 |
| **Lactate at admission (n=772)** | 2.4±1.9 |
| **Base Excess (BE) at admission (n=771)** | -2.5±4.5 |
| **Fluid Balance within 24 h (n=794)** | 679.8±1339.7 |
| **Systolic Blood Pressure at admission (n=797)** |  |
| <90 | 74 (9.3%) |
| ≥90 | 723 (90.7%) |
| **Blood transfusion within 24 h** | |
| Packed red blood cells (PRBC) | 116(14.5 %) |
| Fresh Frozen Plasma (FFP) | 44(5.5%) |
| Platelets | 34(4.3%) |
| Tranexamic Acid (TXA) | 195 (24.5%) |
| Prothrombin complex concentrates (PCC) | 10 (1.3%) |
| Fibrinogen concentrate | 127(15.9%) |
